# Supplementary material for: Impaired DNA damage response signaling by FUS-NLS mutations leads to neurodegeneration and FUS aggregate formation
Source: Nat Commun. 2018 Jan 23;9:335. doi: 10.1038/s41467-017-02299-1 (PMC5780468; doi:10.1038/s41467-017-02299-1)
Supplement: Supplementary file 1 — Supplementary Information [file 41467_2017_2299_MOESM1_ESM.pdf]

Attachment to manuscript

This file includes:

1. Supplementary Fig. 1 to 12 with legends
2. Supplementary Tables 1 to 17

Other Supplementary Materials for this manuscript includes the following:

Quicktime Supplementary Movies 1 to 13

Fig. S1

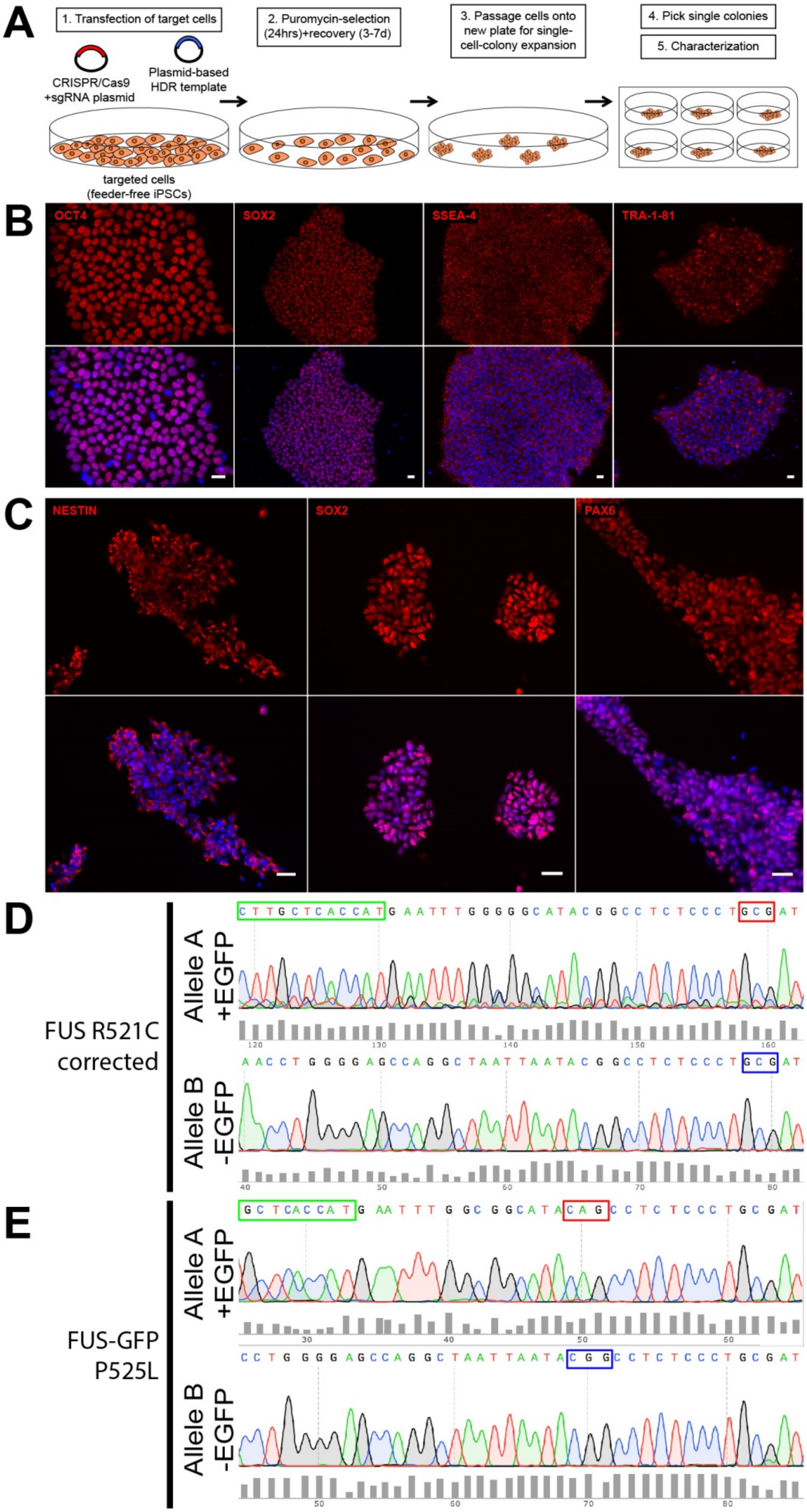

A
1. Transfection of target cells
2. Puromycin-selection (24hrs)+recovery (3-7d)
3. Passage cells onto new plate for single-cell-colony expansion
4. Pick single colonies
5. Characterization
CRISPR/Cas9 +sgRNA plasmid
Plasmid-based HDR template
targeted cells (feeder-free iPSCs)
B
OCT4
SOX2
SSEA-4
TRA-1-81
C
NESTIN
SOX2
PAX6
D
FUS R521C corrected
Allele A +EGFP
Allele B -EGFP
E
FUS-GFP P525L
Allele A +EGFP
Allele B -EGFP

Supplementary Fig. 1: CRISPR/Cas9-mediated genome editing of targeted cells and characterization of isogenic control cell line

(A) Workflow chart of CRISPR/Cas9-mediated genome editing. **(B-C)** Representative images of the generated isogenic control line WT-GFP (Supplementary Table 1) are shown. ICC of iPSCs shows expression of characteristic pluripotency markers OCT4, SOX2, SSEA-4 and TRA-1-81. ICC of NPCs shows expression of characteristic NPC markers NESTIN, SOX2 and PAX6. Nuclei are counter stained with Hoechst, bars: 25µm. **(D)** In order to verify that correction of mutant FUS R521C cell line (Supplementary Table 1) was successful, the genotype of the isogenic control cell line (FUS WT-GFP) was determined by Sanger sequencing. Shown are the reverse strands of both alleles. Allele A shows that FUS mutation at amino acid position 521 (red box) is corrected to WT sequence and EGFP sequence is present (green box). Allele B contains FUS WT sequence at the same position (blue box) **(E)** The same method described was used to generate C-terminal tagged mutant FUS P525L-GFP isogenic iPSCs. Sanger sequencing confirms that allele A possesses the mutant sequence (red box) and allele B the WT sequence (blue box) of FUS at amino acid position 525 (red box).

Fig. S2

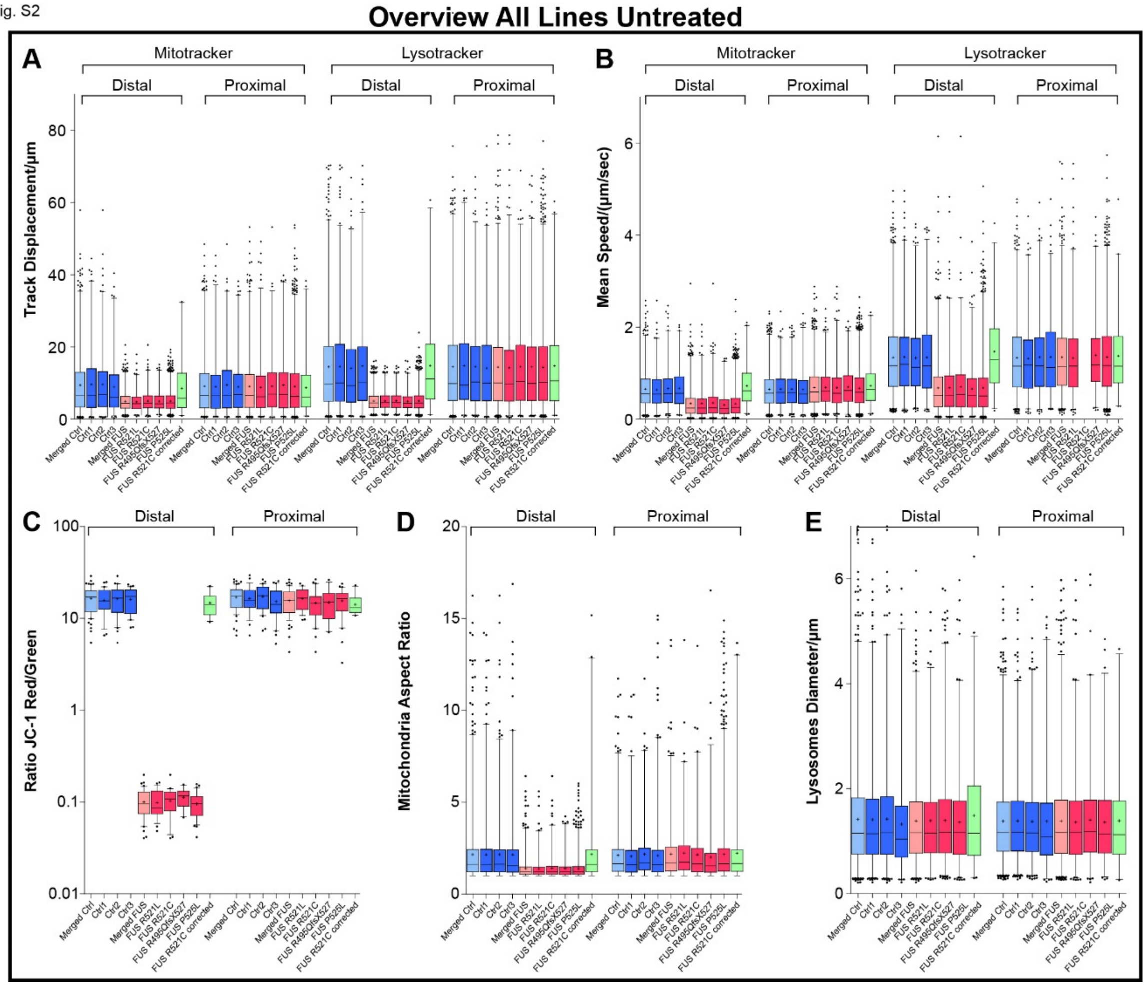

Supplementary Fig. 2

Overview of all control and mutant FUS hiPSC lines as described in Table 1. Shown is the organelle tracking and shape analysis (box plots) as detailed in Methods. Note the phenotypic similarity among all control (Ctrl1-3, in blue) and mutant FUS (FUS R521L, R521C, R495QfsX527, P525L, in red) lines, thereby validating our batch analysis of Ctrl1-3 (Merged Ctrl, in pale blue) and FUS R521L, R521C and R495QfsX527 (Merged FUS, in pale red). Moreover, note the complete distal rescue in the corrected FUS R521C line (in green).

Fig. S3

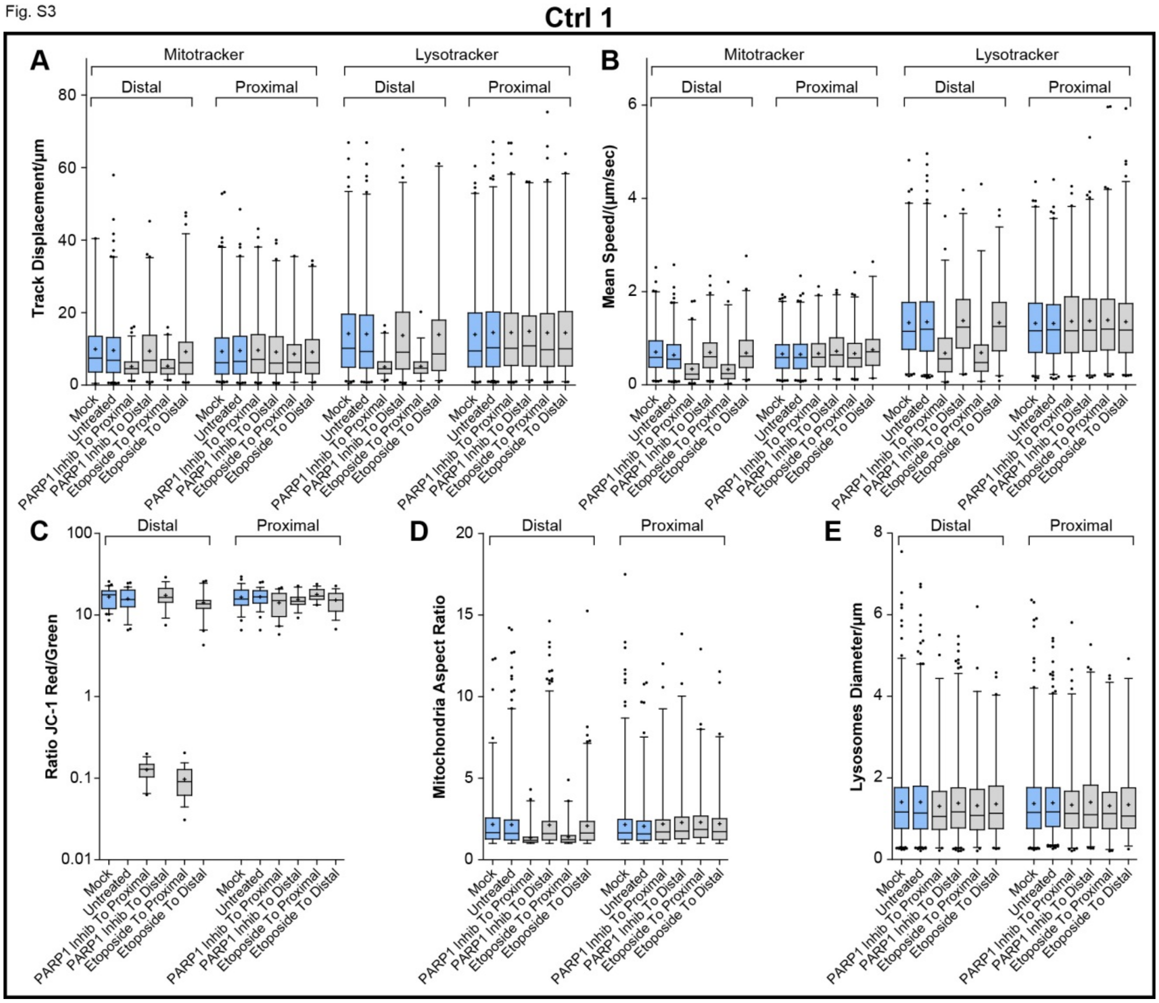

Ctrl 1
A
B
C
D
E
Mitotracker
Lysotracker
Distal
Proximal
Track Displacement/µm
Mean Speed/(µm/sec)
Ratio JC-1 Red/Green
Mitochondria Aspect Ratio
Lysosomes Diameter/µm
Mock
Untreated
PARP1 Inhib To Proximal
PARP1 Inhib To Distal
Etoposide To Proximal
Etoposide To Distal

36

Fig. S4

Ctrl 2

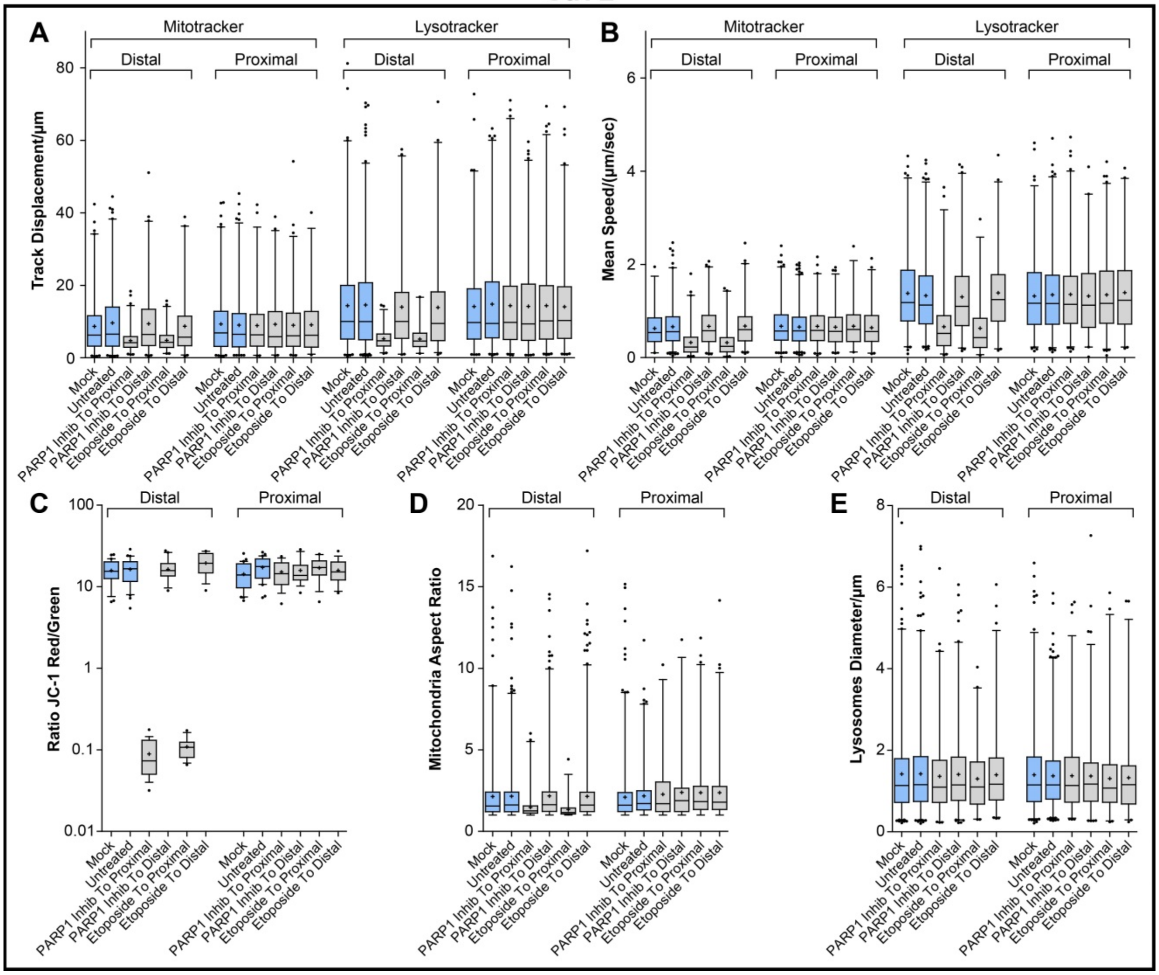
A
Mitotracker
Lysotracker
Distal
Proximal
Track Displacement/µm
Mock
Untreated
PARP1 Inhib To Proximal
PARP1 Inhib To Distal
Etoposide To Proximal
Etoposide To Distal
B
Mean Speed/(µm/sec)
C
Ratio JC-1 Red/Green
D
Mitochondria Aspect Ratio
E
Lysosomes Diameter/µm

Fig. S5

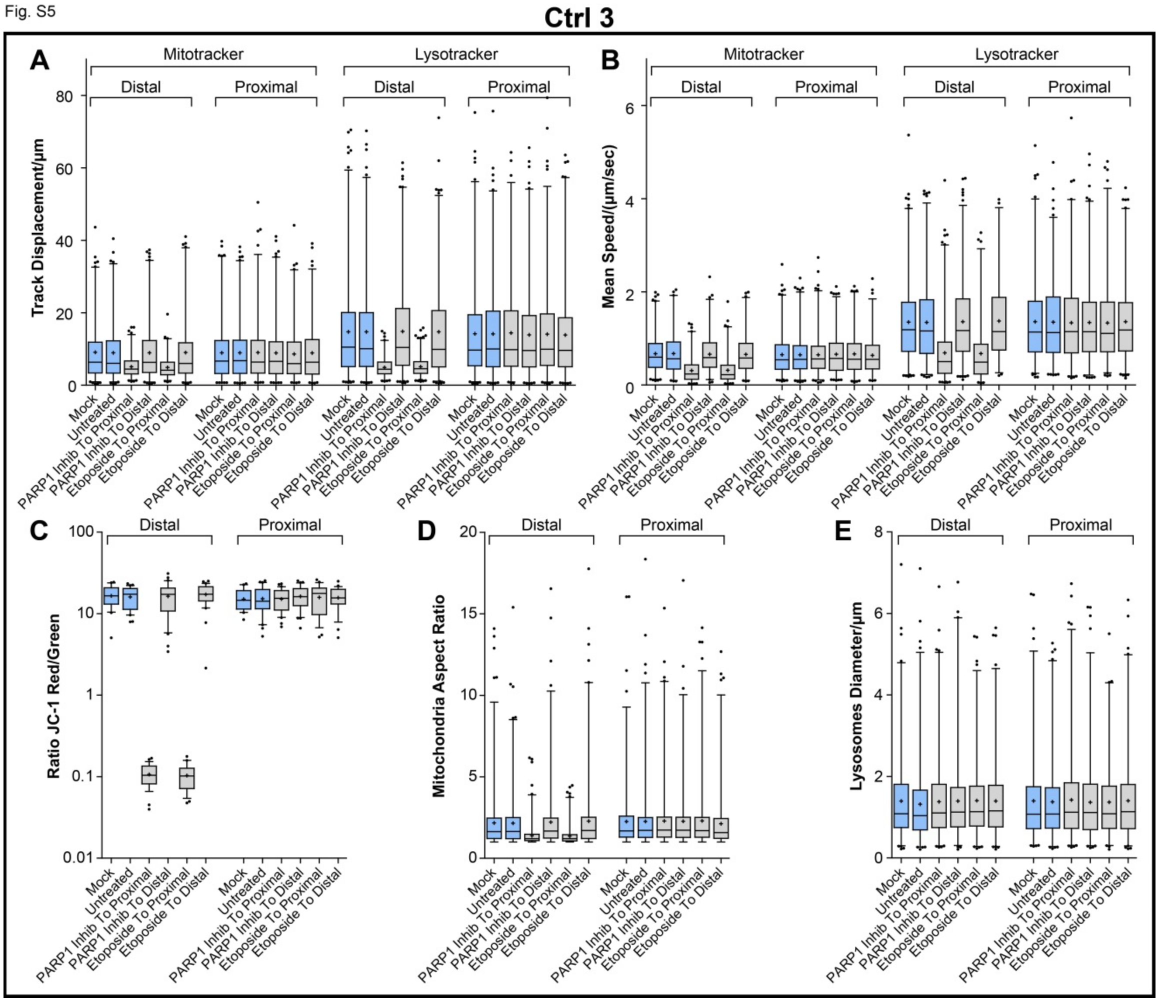
Ctrl 3
A
B
C
D
E
Mitotracker
Lysotracker
Distal
Proximal
Track Displacement/µm
Mean Speed/(µm/sec)
Ratio JC-1 Red/Green
Mitochondria Aspect Ratio
Lysosomes Diameter/µm
Mock
Untreated
PARP1 Inhib To Proximal
PARP1 Inhib To Distal
Etoposide To Proximal
Etoposide To Distal

38

Fig. S6

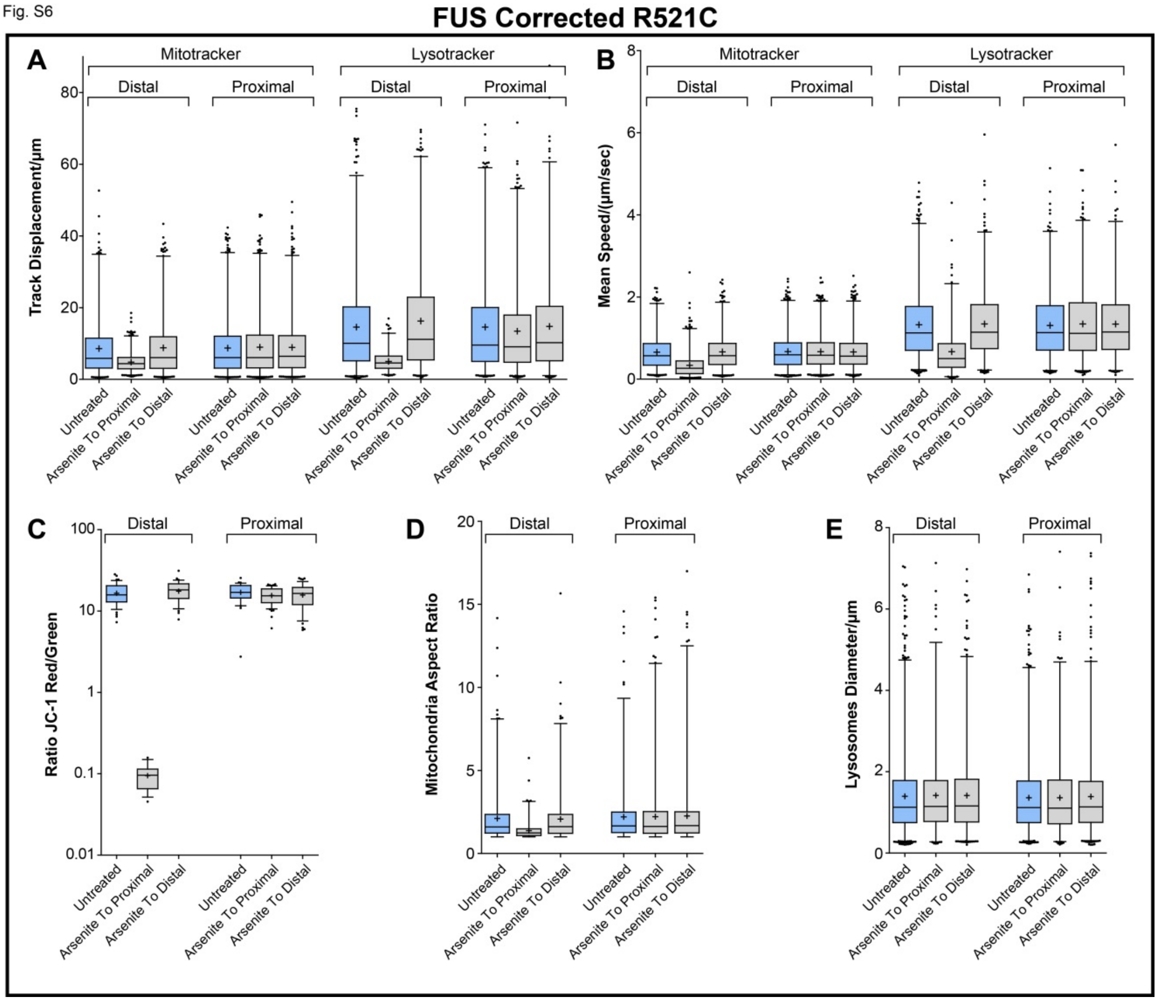

FUS Corrected R521C
A
Mitotracker
Lysotracker
Distal
Proximal
Track Displacement/µm
Untreated
Arsenite To Proximal
Arsenite To Distal
B
Mean Speed/(µm/sec)
C
Ratio JC-1 Red/Green
D
Mitochondria Aspect Ratio
E
Lysosomes Diameter/µm

Fig. S7

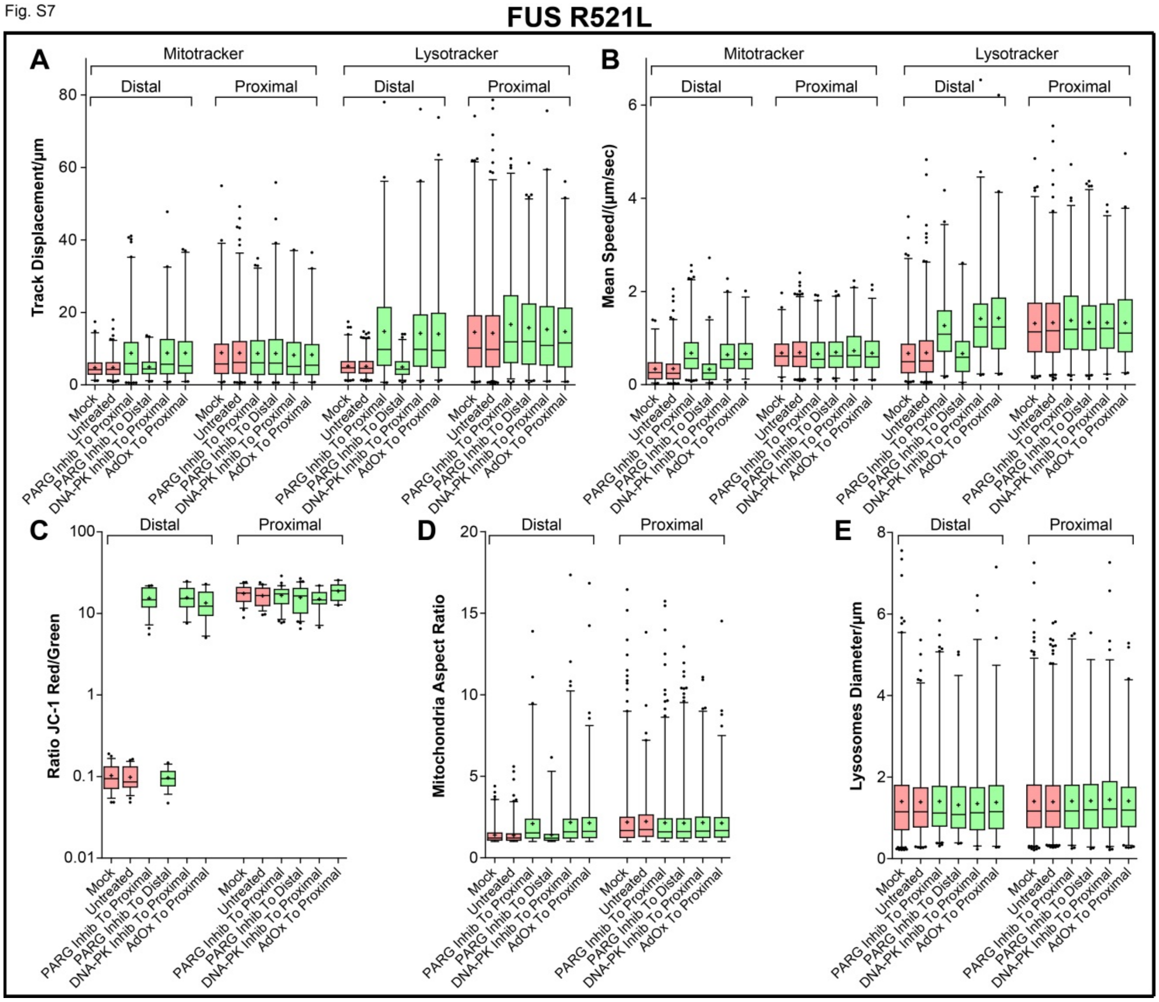
FUS R521L
A
Mitotracker
Lysotracker
Distal
Proximal
Track Displacement/µm
Mock
Untreated
PARG Inhib To Proximal
PARG Inhib To Distal
DNA-PK Inhib To Proximal
AdOx To Proximal
B
Mean Speed/(µm/sec)
C
Ratio JC-1 Red/Green
D
Mitochondria Aspect Ratio
E
Lysosomes Diameter/µm

40

Fig. S8

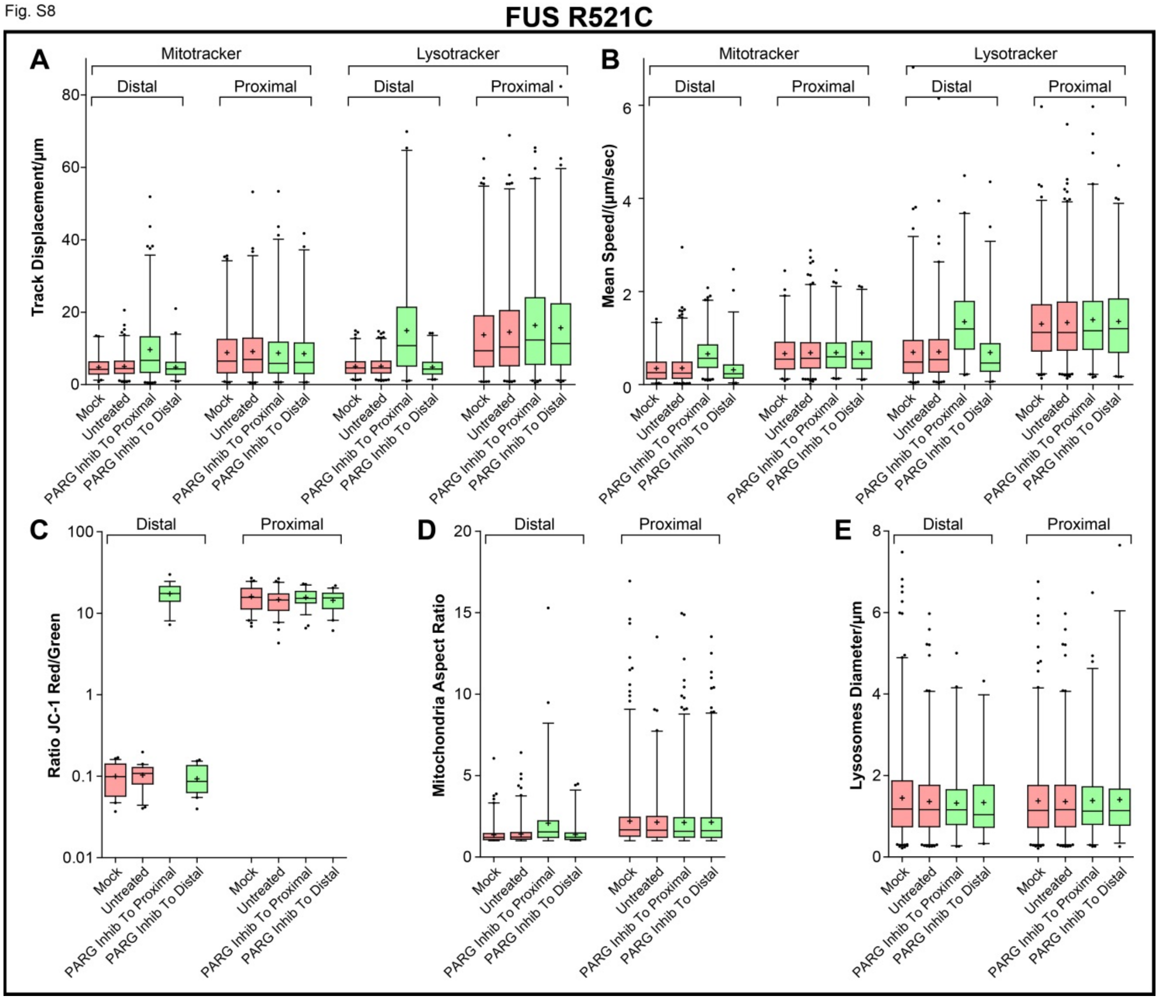
FUS R521C
A
B
C
D
E
Mitotracker
Lysotracker
Distal
Proximal
Track Displacement/µm
Mean Speed/(µm/sec)
Ratio JC-1 Red/Green
Mitochondria Aspect Ratio
Lysosomes Diameter/µm
Mock
Untreated
PARG Inhib To Proximal
PARG Inhib To Distal

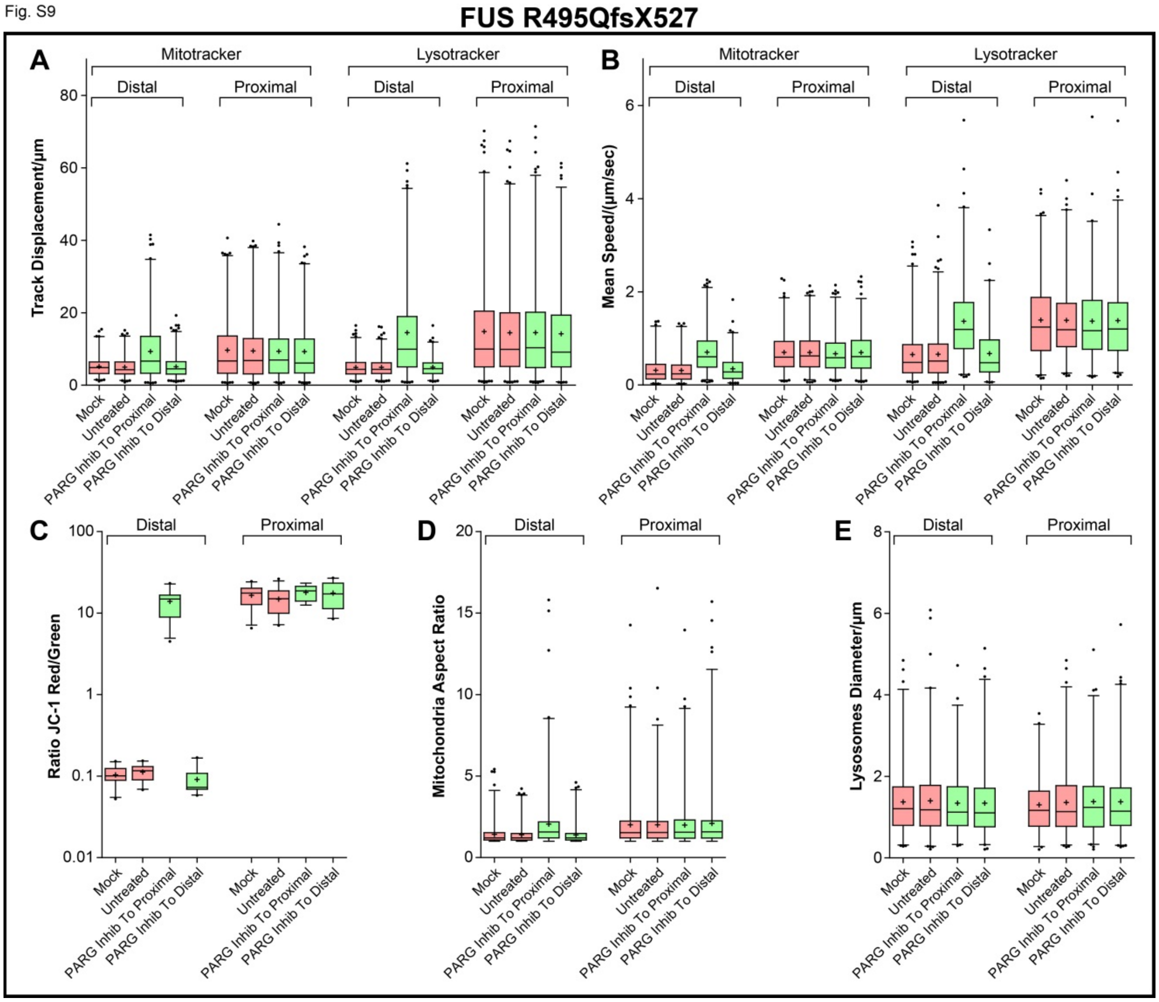

Supplementary Fig. 3-9

Refer to box plots of batch-analyzed iPSC-derived clonal lines in Fig. 4B-C, 4E-I, 5I-M and 6H-L. Shown is the organelle tracking and shape analysis as detailed in Methods of each control (Supplementary Fig. 3-6: Ctrl 1-3, FUS corrected R521C and mutant FUS (Supplementary Fig. 7-9: R521C, R521L, R495QfsX527) line. Note the similarity of phenotypes and compound responses among all control (Supplementary Fig. 3-6) and mutant FUS (Supplementary Fig. 7-9) lines, respectively, justifying our batch analysis in the main manuscript.

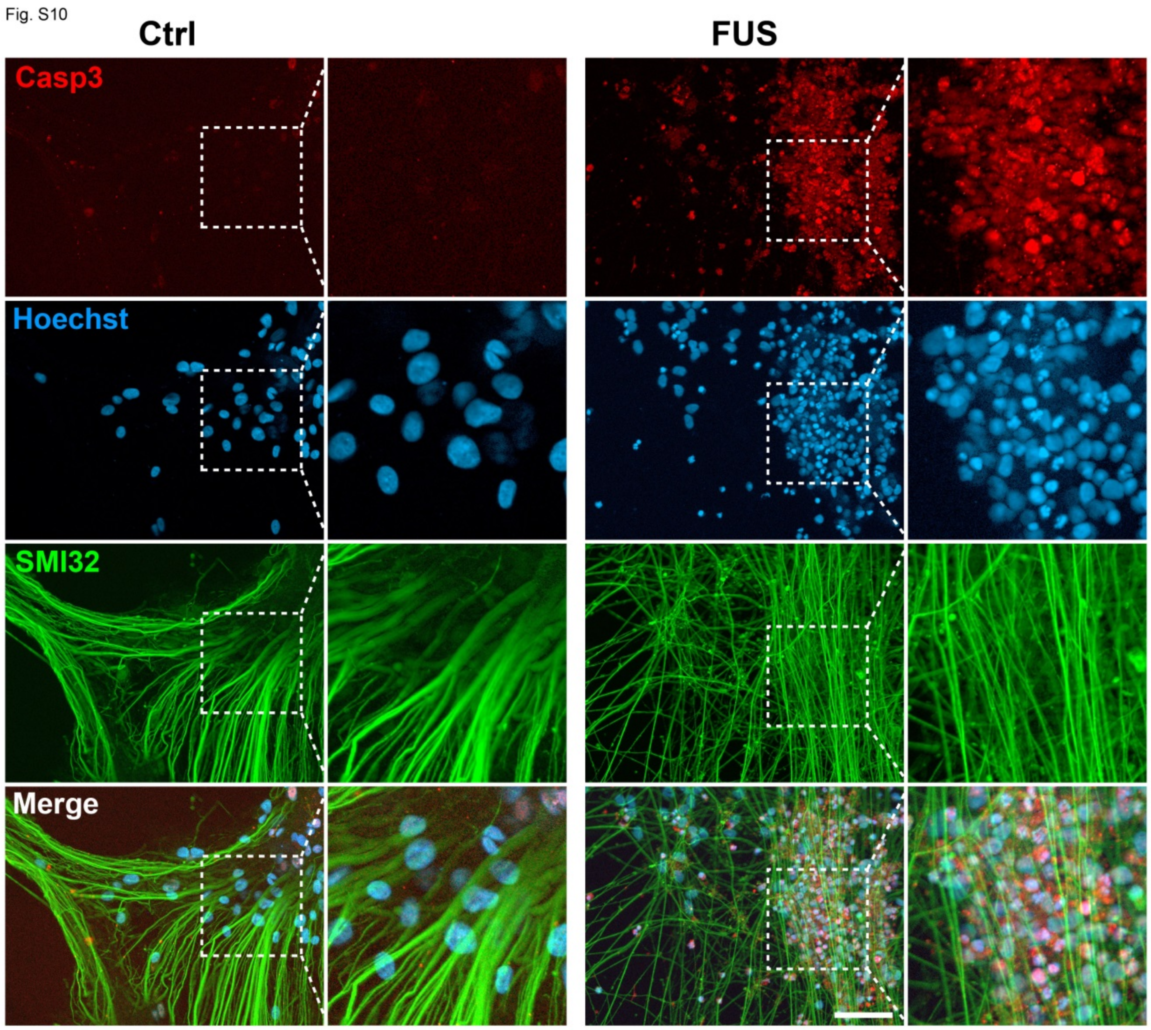

Supplementary Fig. 10

Increased Caspase 3 occurrence (red) in FUS-GFP P525L MN (green, MN marker SMI32) at 110 DIV. Representative examples corresponding to Fig. 2K, bar: 50µm.

Supplementary Fig. 11

full blots of Fig. 1D

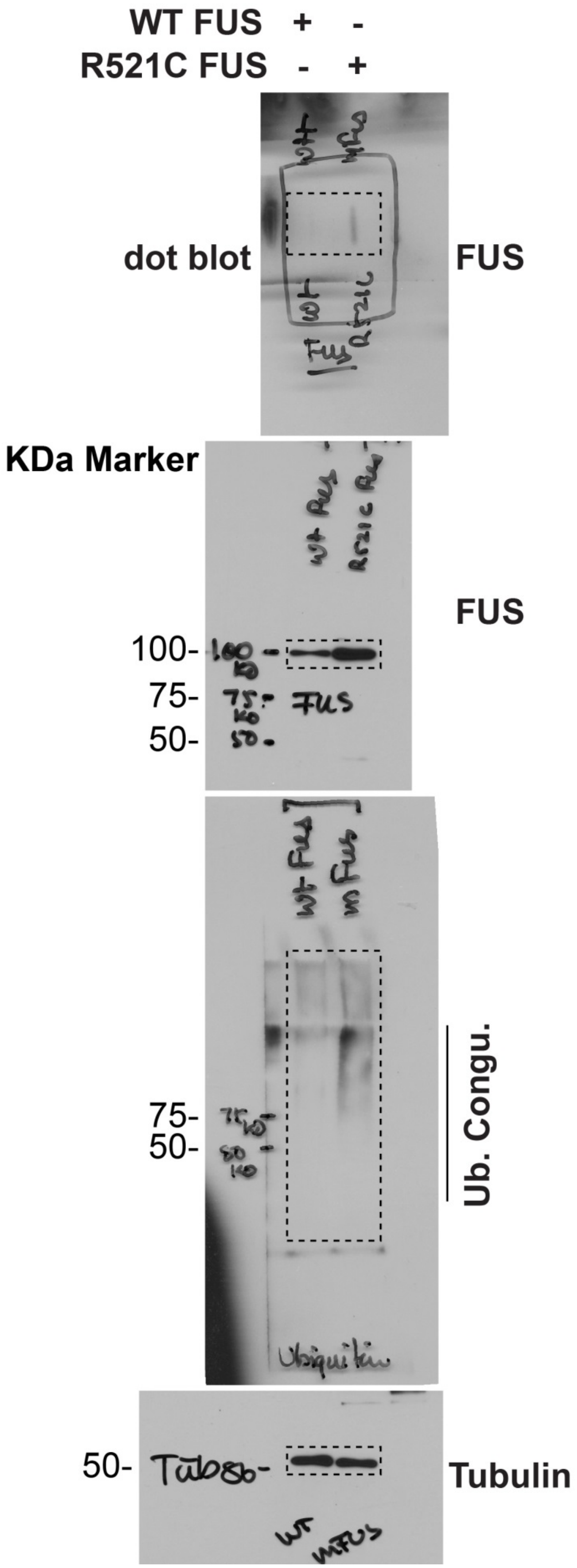

Supplementary Fig. 11

Full length blots corresponding to cropped Western blot panels of Fig. 1D in the main manuscript.

Supplementary Fig. 12

full blots of Fig. 7G

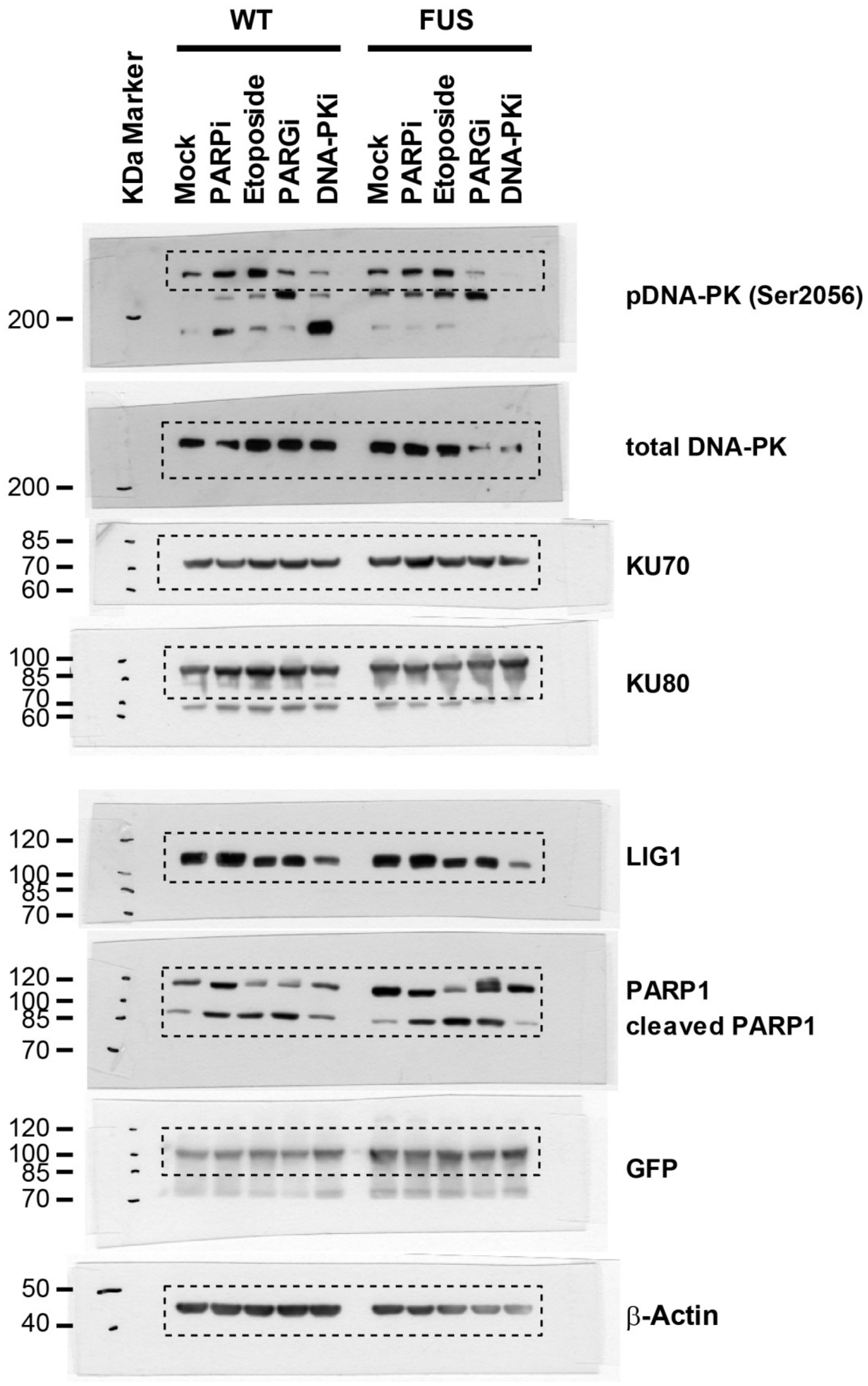

Supplementary Fig. 12

Full length blots corresponding to cropped Western blot panels of Fig. 7G in the main manuscript.

Supplementary Table 1: Box plot statistics of Fig. 4B, track displacement/µm

Box plot statistics of Fig. 4B, track displacement	Mitotracker distal				Lysotracker distal			
	Ctrl 9 DIV	Ctrl 21 DIV	FUS 9 DIV	FUS 21 DIV^{+++}	Ctrl 9 DIV	Ctrl 21 DIV	FUS 9 DIV	FUS 21 DIV^{+++}
Number of values	1151	1151	1908	1292	613	1160	894	979
Minimum	1.2	1.201	1.201	1.2	1.205	1.203	1.204	1.203
25% Percentile	1.855	1.717	1.86	1.643	2.455	2.719	2.428	1.826
Median	2.786	2.464	2.821	2.079	4.904	5.182	4.347	2.544
75% Percentile	4.474	4.359	4.942	2.868	10.29	11.19	10.69	4.357
Maximum	41.83	32.76	99.96	13.92	97.52	81.63	84.06	42.73
Mean	4.171	3.784	4.485	2.536	8.493	9.160	8.747	4.131
Std. Deviation	4.316	3.609	5.203	1.458	10.57	10.67	10.74	4.413
Std. Error	0.1272	0.1431	0.1191	0.04057	0.4268	0.3132	0.3591	0.1410
Lower 95% CI of mean	3.921	3.503	4.251	2.456	7.655	8.545	8.042	3.855
Upper 95% CI of mean	4.421	4.065	4.718	2.615	9.331	9.774	9.451	4.408
Sum	4801	2406	8557	3276	5206	10625	7819	4045

$^{+++}$): highly significant difference to Ctrl 9 DIV with p<0.001, Kruskal-Wallis with Dunns post-hoc test

Supplementary Table 2: Box plot statistics of Fig. 4C, mean speed/(µm/sec)

Box plot statistics of Fig. 4C, mean speed	Mitotracker distal				Lysotracker distal			
	Ctrl 9 DIV	Ctrl 21 DIV	FUS 9 DIV	FUS 21 DIV^{+++}	Ctrl 9 DIV	Ctrl 21 DIV	FUS 9 DIV	FUS 21 DIV^{+++}
Number of values	1151	636	1908	1292	613	1160	894	979
Minimum	0.05658	0.04964	0.04694	0.04152	0.08602	0.09657	0.08522	0.04471
25% Percentile	0.3266	0.2963	0.3559	0.1574	0.4792	0.5348	0.4869	0.1998
Median	0.5586	0.5348	0.5866	0.2638	0.8858	0.9245	0.9086	0.3476
75% Percentile	0.9300	0.9341	0.9587	0.4430	1.635	1.605	1.527	0.5764
Maximum	5.665	3.348	3.893	1.366	5.797	5.894	5.866	2.213
Mean	0.7019	0.6992	0.7202	0.3240	1.195	1.195	1.158	0.4314
Std. Deviation	0.5379	0.5310	0.5097	0.2223	0.9739	0.9099	0.9224	0.3116
Std. Error	0.01585	0.02106	0.01167	0.006183	0.03934	0.02672	0.03085	0.009960
Lower 95% CI of mean	0.6708	0.6578	0.6973	0.3118	1.118	1.143	1.098	0.4118
Upper 95% CI of mean	0.7330	0.7405	0.7431	0.3361	1.272	1.248	1.219	0.4509
Sum	807.9	444.7	1374	418.6	732.6	1387	1035	422.3

$^{+++}$): highly significant difference to Ctrl 9 DIV with p<0.001, Kruskal-Wallis with Dunns post-hoc test

Supplementary Table 3: Box plot statistics of Fig. 4E, track displacement/µm

Box plot statistics of Fig. 4E, track displacement	Mitotracker						Lysotracker					
	Distal			*Proximal*			*Distal*			*Proximal*		
	Ctrl	FUS***	FUS corrected	Ctrl	FUS	FUS corrected	Ctrl	FUS***	FUS corrected	Ctrl	FUS	FUS corrected
**Number of values**	1697	1553	194	1502	1545	226	1957	2057	183	1755	1885	234
**Minimum**	0.3651	0.6395	0.8860	0.4324	0.4462	0.8226	0.4884	1.012	1.169	0.4454	0.3569	1.009
**25% Percentile**	3.357	3.064	3.179	3.134	3.252	3.426	4.936	3.227	5.650	5.168	5.104	5.101
**Median**	6.503	4.351	5.803	6.617	6.625	6.062	9.789	4.505	11.15	9.859	10.01	10.61
**75% Percentile**	13.01	6.314	12.75	12.63	12.39	12.20	20.14	6.38	20.75	20.44	19.9	20.37
**Maximum**	57.92	20.56	32.53	48.48	53.21	38.30	70.33	16.19	60.66	92.44	78.63	60.31
**Mean**	9.409	4.921	8.491	9.13	9.088	8.714	14.49	5.033	14.83	14.49	14.39	14.74
**Std. Deviation**	8.209	2.566	7.424	8.089	8.029	7.527	12.92	2.515	12.58	12.74	12.64	12.65
**Std. Error**	0.1993	0.06512	0.5330	0.2087	0.2043	0.5007	0.292	0.05546	0.9299	0.3041	0.2912	0.8268
**Lower 95% CI of mean**	9.018	4.793	7.440	8.72	8.687	7.728	13.91	4.925	12.99	13.89	13.82	13.11
**Upper 95% CI of mean**	9.8	5.049	9.542	9.539	9.489	9.701	15.06	5.142	16.66	15.08	14.96	16.37
**Sum**	15967	7643	1647	13713	14041	1969	28347	10354	2713	25421	27130	3448

***): highly significant difference to all other conditions with $p<0.001$, Kruskal-Wallis with Dunns post-hoc test

Supplementary Table 4: Box plot statistics of Fig. 4F, mean speed/(µm/sec)

Box plot statistics of Fig. 4F, mean speed	Mitotracker						Lysotracker					
	Distal			*Proximal*			*Distal*			*Proximal*		
	Ctrl	FUS***	FUS corrected	Ctrl	FUS	FUS corrected	Ctrl	FUS***	FUS corrected	Ctrl	FUS	FUS corrected
**Number of values**	1614	1911	242	1997	2304	209	2031	2057	166	1646	1874	220
**Minimum**	0.043	0.00835	0.09408	0.05541	0.01711	0.09604	0.1528	0.0388	0.1871	0.1204	0.09846	0.2822
**25% Percentile**	0.3566	0.1297	0.3955	0.3545	0.3748	0.4060	0.7153	0.2719	0.7926	0.6986	0.7372	0.7899
**Median**	0.5552	0.2444	0.6185	0.5673	0.5942	0.6488	1.163	0.5203	1.289	1.152	1.146	1.154
**75% Percentile**	0.8723	0.4534	0.9987	0.8631	0.9225	0.9842	1.787	0.9198	1.965	1.783	1.758	1.796
**Maximum**	2.578	2.949	2.103	2.34	2.882	2.322	4.961	6.144	4.257	4.78	5.59	4.779
**Mean**	0.6555	0.3411	0.7213	0.6519	0.6884	0.7262	1.341	0.6806	1.471	1.338	1.344	1.375
**Std. Deviation**	0.3971	0.2994	0.4177	0.3885	0.4167	0.4387	0.8087	0.5757	0.8358	0.8123	0.8171	0.7563
**Std. Error**	0.00989	0.00685	0.02685	0.00869	0.00868	0.03035	0.01794	0.01269	0.06487	0.02002	0.01887	0.05099
**Lower 95% CI of mean**	0.6361	0.3277	0.6684	0.6348	0.6713	0.6664	1.306	0.6558	1.343	1.299	1.307	1.275
**Upper 95% CI of mean**	0.6749	0.3546	0.7742	0.6689	0.7054	0.7860	1.376	0.7055	1.599	1.377	1.381	1.476
**Sum**	1058	651.9	174.6	1302	1586	151.8	2724	1400	244.2	2202	2518	302.5

***): highly significant difference to all other conditions with $p<0.001$, Kruskal-Wallis with Dunns post-hoc test

Supplementary Table 5: Box plot statistics of Fig. 4G, ratio JC-1 red/green

Box plot statistics of Fig. 4G, ratio JC-1 red/green	Mitotracker JC-1					
	Distal			*Proximal*		
	Ctrl	FUS***	FUS corrected	Ctrl	FUS	FUS corrected
**Number of values**	60	46	10	59	58	10
**Minimum**	5.434	0.04023	9.089	6.514	4.308	10.72
**25% Percentile**	11.83	0.07482	10.98	13.15	11.53	11.56
**Median**	17.1	0.09567	14.07	17.41	15.83	13.11
**75% Percentile**	19.95	0.1282	17.41	20.53	19.39	16.59
**Maximum**	28.93	0.1976	22.44	26.51	26.59	22.87
**Mean**	16.49	0.1005	14.79	16.98	15.56	14.22
**Std. Deviation**	5.133	0.03518	4.170	4.767	5.034	3.813
**Std. Error**	0.6626	0.00519	1.319	0.6206	0.661	1.206
**Lower 95% CI of mean**	15.17	0.09003	11.81	15.73	14.23	11.49
**Upper 95% CI of mean**	17.82	0.1109	17.77	18.22	16.88	16.94
**Sum**	989.5	4.622	147.9	1002	902.2	142.2

***): highly significant difference to all other conditions with $p<0.001$, Kruskal-Wallis with Dunns post-hoc test

Supplementary Table 6: Box plot statistics of Fig. 4H, mitochondria aspect ratio

Box plot statistics of Fig. 4H, mitochondria aspect ratio	Mitotracker					
	Distal			*Proximal*		
	Ctrl	FUS***	FUS corrected	Ctrl	FUS	FUS corrected
**Number of values**	2179	1344	214	1075	836	228
**Minimum**	1	1	1.000	1	1	1.000
**25% Percentile**	1.211	1.09	1.221	1.247	1.261	1.244
**Median**	1.621	1.219	1.612	1.657	1.695	1.665
**75% Percentile**	2.425	1.479	2.406	2.428	2.555	2.399
**Maximum**	16.24	6.416	15.17	11.72	13.83	13.66
**Mean**	2.147	1.393	2.163	2.113	2.183	2.219
**Std. Deviation**	1.588	0.5385	1.841	1.353	1.423	1.907
**Std. Error**	0.03402	0.01469	0.1258	0.04127	0.04921	0.1263
**Lower 95% CI of mean**	2.08	1.364	1.915	2.032	2.086	1.971
**Upper 95% CI of mean**	2.214	1.422	2.411	2.194	2.279	2.468
**Sum**	4678	1872	462.9	2271	1825	506.0

***): highly significant difference to all other conditions with p<0.001, Kruskal-Wallis with Dunns post-hoc test

Supplementary Table 7: Box plot statistics of Fig. 4I, lysosomes diameter/µm

Box plot statistics of Fig. 4I, lysosomes diameter	Lysotracker					
	Distal			***Proximal***		
	**Ctrl**	**FUS**	**FUS corrected**	**Ctrl**	**FUS**	**FUS corrected**
**Number of values**	2173	1135	219	2153	1882	137
**Minimum**	0.2063	0.2113	0.2938	0.2662	0.2584	0.2519
**25% Percentile**	0.7486	0.7704	0.7268	0.8067	0.7621	0.7466
**Median**	1.146	1.157	1.148	1.163	1.165	1.119
**75% Percentile**	1.823	1.747	2.051	1.743	1.779	1.762
**Maximum**	6.999	6.151	6.423	5.847	5.973	4.660
**Mean**	1.413	1.382	1.486	1.378	1.38	1.388
**Std. Deviation**	0.9375	0.8634	1.031	0.8102	0.8643	0.8874
**Std. Error**	0.02011	0.02563	0.06970	0.01746	0.01992	0.07581
**Lower 95% CI of mean**	1.374	1.332	1.348	1.344	1.341	1.238
**Upper 95% CI of mean**	1.453	1.432	1.623	1.412	1.419	1.538
**Sum**	3071	1569	325.4	2967	2597	190.1

Supplementary Table 8: Box plot statistics of Fig. 5I, track displacement/µm

Box plot statistics of Fig. 5I, track displacement	Mitotracker distal				Lysotracker distal			
	Ctrl Mock	FUS Mock***	Ctrl + Etoposide***	Ctrl + Arsenite***	Ctrl Mock	FUS Mock***	Ctrl + Etoposide***	Ctrl + Arsenite***
**Number of values**	1123	942	817	1226	1532	1512	1154	652
**Minimum**	0.3881	0.7597	0.8897	0.7753	0.4833	0.9128	1.009	1.007
**25% Percentile**	3.369	3.089	2.958	2.961	5.078	3.205	3.221	3.083
**Median**	6.368	4.483	4.301	4.367	10.25	4.528	4.573	4.547
**75% Percentile**	12.21	6.313	6.423	6.109	19.98	6.304	6.501	6.478
**Maximum**	43.6	17.44	19.62	18.52	81.22	17.42	20.23	16.96
**Mean**	9.096	4.933	4.96	4.814	14.48	5.014	5.111	4.994
**Std. Deviation**	7.865	2.49	2.618	2.461	12.59	2.488	2.548	2.508
**Std. Error**	0.2347	0.08112	0.09158	0.07029	0.3217	0.06399	0.075	0.09820
**Lower 95% CI of mean**	8.636	4.774	4.78	4.676	13.84	4.888	4.964	4.802
**Upper 95% CI of mean**	9.557	5.092	5.14	4.952	15.11	5.139	5.258	5.187
**Sum**	10215	4647	4052	5902	22176	7581	5898	3256

***): highly significant difference to Ctrl Mock with p<0.001, Kruskal-Wallis with Dunns post-hoc test

Supplementary Table 9: Box plot statistics of Fig. 5J, mean speed/(µm/sec)

Box plot statistics of Fig. 5J, mean speed	Mitotracker distal				Lysotracker distal			
	Ctrl Mock	FUS Mock***	Ctrl + Etoposide***	Ctrl + Arsenite***	Ctrl Mock	FUS Mock***	Ctrl + Etoposide ***	Ctrl + Arsenite***
**Number of values**	1123	920	835	1226	1547	1484	767	652
**Minimum**	0.0732	0.01469	0.02222	0.02699	0.08042	0.03584	0.03892	0.02654
**25% Percentile**	0.3729	0.1274	0.1279	0.1345	0.7508	0.2567	0.2432	0.2901
**Median**	0.5827	0.2507	0.2352	0.2661	1.176	0.4874	0.4751	0.5026
**75% Percentile**	0.9041	0.4672	0.4287	0.4440	1.798	0.882	0.8675	0.8639
**Maximum**	2.52	1.409	2.21	2.600	5.365	6.816	4.307	4.296
**Mean**	0.6753	0.3312	0.3208	0.3384	1.354	0.6713	0.6668	0.6690
**Std. Deviation**	0.3983	0.271	0.2753	0.2729	0.8107	0.5894	0.5965	0.5434
**Std. Error**	0.01189	0.008935	0.009527	0.007793	0.02061	0.0153	0.02154	0.02128
**Lower 95% CI of mean**	0.652	0.3136	0.3021	0.3231	1.313	0.6413	0.6245	0.6272
**Upper 95% CI of mean**	0.6986	0.3487	0.3395	0.3536	1.394	0.7013	0.7091	0.7108
**Sum**	758.3	304.7	267.9	414.8	2094	996.3	511.4	436.2

***): highly significant difference to Ctrl Mock with $p<0.001$, Kruskal-Wallis with Dunns post-hoc test

Supplementary Table 10: Box plot statistics of Fig. 5K, ratio JC-1 red/green

Box plot statistics of Fig. 5K, ratio JC-1 red/green	Mitotracker JC-1 distal			
	Ctrl Mock	FUS Mock***	Ctrl + Etoposide***	Ctrl + Arsenite***
Number of values	55	48	36	18
Minimum	6.537	0.03684	0.03083	0.04543
25% Percentile	12.37	0.06978	0.07374	0.06559
Median	15.75	0.09657	0.09835	0.09599
75% Percentile	20.12	0.139	0.1253	0.1141
Maximum	26.27	0.1902	0.204	0.1575
Mean	16.02	0.1011	0.1026	0.09501
Std. Deviation	5.053	0.04103	0.03861	0.03255
Std. Error	0.6813	0.005922	0.006435	0.007672
Lower 95% CI of mean	14.66	0.08917	0.08949	0.07883
Upper 95% CI of mean	17.39	0.113	0.1156	0.1112
Sum	881.3	4.852	3.692	1.710

***): highly significant difference to Ctrl Mock with p<0.001, Kruskal-Wallis with Dunns post-hoc test

Supplementary Table 11: Box plot statistics of Fig. 5L, mitochondria aspect ratio

Box plot statistics of Fig. 5L, mitochondria aspect ratio	Mitotracker distal			
	Ctrl Mock	FUS Mock***	Ctrl + Etoposide***	Ctrl + Arsenite***
**Number of values**	1143	917	220	504
**Minimum**	1	1	1	1
**25% Percentile**	1.241	1.077	1.079	1.081
**Median**	1.61	1.211	1.229	1.235
**75% Percentile**	2.427	1.478	1.486	1.487
**Maximum**	16.88	6.055	4.889	5.746
**Mean**	2.151	1.377	1.395	1.375
**Std. Deviation**	1.572	0.496	0.5194	0.4754
**Std. Error**	0.04651	0.01638	0.03502	0.02118
**Lower 95% CI of mean**	2.06	1.345	1.326	1.334
**Upper 95% CI of mean**	2.242	1.409	1.464	1.417
**Sum**	2459	1263	307	693.2

***): highly significant difference to Ctrl Mock with $p<0.001$, Kruskal-Wallis with Dunns post-hoc test

Supplementary Table 12: Box plot statistics of Fig. 5M, lysosomes diameter/µm

Box plot statistics of Fig. 5M, lysosomes diameter	Lysotracker distal			
	Ctrl Mock	FUS Mock	Ctrl + Etoposide	Ctrl + Arsenite***
Number of values	1769	1804	496	683
Minimum	0.2173	0.2169	0.2108	0.2339
25% Percentile	0.7427	0.729	0.716	0.7759
Median	1.151	1.166	1.09	1.147
75% Percentile	1.781	1.831	1.713	1.779
Maximum	7.578	8.319	6.201	7.129
Mean	1.413	1.423	1.31	1.416
Std. Deviation	0.9706	1.007	0.7919	0.9590
Std. Error	0.02308	0.02372	0.03556	0.03670
Lower 95% CI of mean	1.368	1.377	1.24	1.344
Upper 95% CI of mean	1.458	1.47	1.38	1.488
Sum	2499	2567	649.9	967.4

Supplementary Table 13: Box plot statistics of Fig. 6H, track displacement/µm

Box plot statistics of Fig. 6H, track displacement	Mitotracker distal						Lysotracker distal					
	Ctrl Mock	FUS Mock	Ctrl + PARP1 inhib.***	FUS + PAPRG inhib.+++	FUS +DNA-PK inhib.+++	FUS + AdOx+++	Ctrl Mock	FUS Mock	Ctrl + PARP1 inhib.***	FUS + PAPRG inhib.+++	FUS +DNA-PK inhib.+++	FUS + AdOx+++
Number of values	1123	942	1214	1586	282	247	1532	1512	974	1025	225	225
Minimum	0.3881	0.7597	0.8406	0.3117	0.5826	0.8979	0.4833	0.9128	1.025	0.612	0.8017	0.8861
25% Percentile	3.369	3.089	3.059	3.217	3.069	3.231	5.078	3.205	3.199	5.158	5.145	4.828
Median	6.368	4.483	4.388	6.401	5.702	5.271	10.25	4.528	4.557	10.06	9.798	9.504
75% Percentile	12.21	6.313	6.297	12.83	12.52	11.9	19.98	6.304	6.412	20.66	19.29	19.76
Maximum	43.6	17.44	18.38	51.9	47.75	37.39	81.22	17.42	16.49	78.02	76.08	73.78
Mean	9.096	4.933	4.989	9.236	8.793	8.799	14.48	5.014	5.035	14.65	14.24	14.04
Std. Deviation	7.865	2.49	2.631	8.156	7.718	8.406	12.59	2.488	2.504	13	12.62	12.54
Std. Error	0.2347	0.0811	0.0755	0.2048	0.4596	0.5348	0.3217	0.064	0.0802	0.4059	0.841	0.8357
Lower 95% CI of mean	8.636	4.774	4.841	8.834	7.888	7.745	13.84	4.888	4.877	13.85	12.58	12.4
Upper 95% CI of mean	9.557	5.092	5.138	9.638	9.698	9.852	15.11	5.139	5.192	15.44	15.89	15.69
Sum	10215	4647	6057	14649	2480	2173	22176	7581	4904	15013	3203	3160

***): highly significant difference to Ctrl Mock with $p<0.001$, Kruskal-Wallis with Dunns post-hoc test

+++): highly significant difference to FUS Mock with $p<0.001$, Kruskal-Wallis with Dunns post-hoc test

Supplementary Table 14: Box plot statistics of Fig. 6I, mean speed/(µm/sec)

Box plot statistics of Fig. 6I, mean speed	Mitotracker distal						Lysotracker distal					
	Ctrl Mock	FUS Mock	Ctrl + PARP1 inhib.***	FUS + PAPRG inhib.+++	FUS +DNA-PK inhib.+++	FUS + AdOx+++	Ctrl Mock	FUS Mock	Ctrl + PARP1 inhib.***	FUS + PAPRG inhib.+++	FUS +DNA-PK inhib.+++	FUS + AdOx+++
Number of values	1123	920	1142	1642	226	195	1547	1484	1158	959	219	255
Minimum	0.0732	0.0147	0.0245	0.062	0.0564	0.0479	0.0804	0.0358	0.0479	0.1559	0.2019	0.2195
25% Percentile	0.3729	0.1274	0.1208	0.3661	0.3534	0.3459	0.7508	0.2567	0.2569	0.751	0.8109	0.7731
Median	0.5827	0.2507	0.2315	0.5815	0.5418	0.5453	1.176	0.4874	0.5178	1.166	1.236	1.236
75% Percentile	0.9041	0.4672	0.4388	0.9047	0.864	0.8781	1.798	0.882	0.919	1.751	1.73	1.856
Maximum	2.52	1.409	1.801	2.561	2.274	2.01	5.365	6.816	4.394	5.685	6.539	6.213
Mean	0.6753	0.3312	0.3237	0.6814	0.6416	0.6639	1.354	0.6713	0.6819	1.338	1.414	1.428
Std. Deviation	0.3983	0.271	0.2738	0.4201	0.4093	0.421	0.8107	0.5894	0.5827	0.7878	0.8496	0.8699
Std. Error	0.0119	0.0089	0.0081	0.0104	0.0272	0.0302	0.0206	0.0153	0.0171	0.0254	0.0574	0.0545
Lower 95% CI of mean	0.652	0.3136	0.3078	0.661	0.588	0.6044	1.313	0.6413	0.6483	1.288	1.301	1.321
Upper 95% CI of mean	0.6986	0.3487	0.3396	0.7017	0.6953	0.7233	1.394	0.7013	0.7155	1.388	1.527	1.535
Sum	758.3	304.7	369.6	1119	145	129.5	2094	996.3	789.6	1283	309.7	364.2

***): highly significant difference to Ctrl Mock with p<0.001, Kruskal-Wallis with Dunns post-hoc test

+++): highly significant difference to FUS Mock with p<0.001, Kruskal-Wallis with Dunns post-hoc test

Supplementary Table 15: Box plot statistics of Fig. 6J, ratio JC-1 red/green

Box plot statistics of Fig. 6J, ratio JC-1 red/green	Mitotracker JC-1 distal					
	Ctrl Mock	FUS Mock	Ctrl + PARP1 inhib.***	FUS + PAPRG inhib.+++	FUS + DNA-PK inhib.+++	FUS + AdOx+++
Number of values	55	48	34	38	10	10
Minimum	6.537	0.03684	0.03179	5.553	8.61	4.577
25% Percentile	12.37	0.06978	0.06213	12.6	10.57	9.43
Median	15.75	0.09657	0.1087	16.98	16.65	12.42
75% Percentile	20.12	0.139	0.1358	21.07	19.49	18.82
Maximum	26.27	0.1902	0.1988	29.86	23.25	20.92
Mean	16.02	0.1011	0.1057	16.2	15.64	13.46
Std. Deviation	5.053	0.04103	0.04429	5.261	5.139	5.378
Std. Error	0.6813	0.005922	0.007595	0.8534	1.625	1.701
Lower 95% CI of mean	14.66	0.08917	0.09024	14.47	11.96	9.616
Upper 95% CI of mean	17.39	0.113	0.1211	17.93	19.32	17.31
Sum	881.3	4.852	3.594	615.7	156.4	134.6

***): highly significant difference to Ctrl Mock with p<0.001, Kruskal-Wallis with Dunns post-hoc test

+++): highly significant difference to FUS Mock with p<0.001, Kruskal-Wallis with Dunns post-hoc test

Supplementary Table 16: Box plot statistics of Fig. 6K, mitochondria aspect ratio

Box plot statistics of Fig. 6K, mitochondria aspect ratio	Mitotracker distal					
	Ctrl Mock	FUS Mock	Ctrl + PARP1 inhib.***	FUS + PAPRG inhib.$^{+++}$	FUS + DNA-PK inhib.$^{+++}$	FUS + AdOx^{+++}
Number of values	1143	917	447	573	460	451
Minimum	1	1	1	1	1	1
25% Percentile	1.241	1.077	1.097	1.211	1.214	1.237
Median	1.61	1.211	1.223	1.534	1.592	1.623
75% Percentile	2.427	1.478	1.465	2.306	2.36	2.471
Maximum	16.88	6.055	6.011	15.29	17.34	16.83
Mean	2.151	1.377	1.403	2.09	2.161	2.141
Std. Deviation	1.572	0.496	0.5813	1.563	1.754	1.569
Std. Error	0.04651	0.01638	0.02749	0.06528	0.08178	0.07386
Lower 95% CI of mean	2.06	1.345	1.349	1.961	2.001	1.996
Upper 95% CI of mean	2.242	1.409	1.457	2.218	2.322	2.286
Sum	2459	1263	627	1197	994.2	965.5

***): highly significant difference to Ctrl Mock with $p<0.001$, Kruskal-Wallis with Dunns post-hoc test

+++): highly significant difference to FUS Mock with $p<0.001$, Kruskal-Wallis with Dunns post-hoc test

Supplementary Table 17: Box plot statistics of Fig. 6L, lysosomes diameter/µm

Box plot statistics of Fig. 6L, lysosomes diameter/µm	Lysotracker distal					
	Ctrl Mock	FUS Mock	Ctrl + PARP1 inhib.	FUS + PAPRG inhib.	FUS + DNA-PK inhib.	FUS + AdOx
Number of values	1769	1804	574	810	235	264
Minimum	0.2173	0.2169	0.2203	0.26	0.233	0.2794
25% Percentile	0.7427	0.729	0.7241	0.7978	0.7088	0.7367
Median	1.151	1.166	1.072	1.137	1.125	1.148
75% Percentile	1.781	1.831	1.711	1.729	1.738	1.781
Maximum	7.578	8.319	6.456	5.841	6.452	7.15
Mean	1.413	1.423	1.335	1.376	1.35	1.376
Std. Deviation	0.9706	1.007	0.863	0.8534	0.8994	0.8937
Std. Error	0.02308	0.02372	0.03602	0.02998	0.05867	0.055
Lower 95% CI of mean	1.368	1.377	1.264	1.317	1.235	1.268
Upper 95% CI of mean	1.458	1.47	1.406	1.435	1.466	1.484
Sum	2499	2567	766.2	1115	317.3	363.2
