# Supplementary material for: Impaired DNA damage response signaling by FUS-NLS mutations leads to neurodegeneration and FUS aggregate formation
Source: Nat Commun. 2018 Jan 23;9:335. doi: 10.1038/s41467-017-02299-1 (PMC5780468; doi:10.1038/s41467-017-02299-1)
Supplement: Supplementary file 3 — Description of Additional Supplementary Files [file 41467_2017_2299_MOESM3_ESM.pdf]

Description of Additional Supplementary Files

File Name: Supplementary Movie 1
Description: Refers to Fig. 4A (showing maximum intensity projections of Mitotracker Deep Red FM), left, and Lysotracker Red DND-99, right). Shows mitochondria trafficking in short 150µm-MFCs to enable imaging at distal axons at earlier time points (i.e. 9 DIV) as the standard length of 900µm requires at least 14 DIV for full channel penetration. Note the distal loss of motility in FUS at 21 DIV (bottom) as opposed to normal trafficking at 9 DIV.

File Name: Supplementary Movie 2
Description: Same as for Supplementary Movie 1 but for lysosomes.

File Name: Supplementary Movie 3
Description: Refers to Fig. 4D (showing maximum intensity projections of Mitotracker Deep Red FM, top, Lysotracker Red DND-99, mid, and first movie frames of Mitotracker JC-1, bottom). Note the distal loss of motility and mitochondrial membrane potential in FUS and the rescue in the isogenic control (FUS corrected).

File Name: Supplementary Movie 4
Description: Refers to Fig. 5H (showing maximum intensity projections of Mitotracker JC-1, left, and Lysotracker Red DND- 99, right), Etoposide or Arsenite addition to Ctrl either exclusively to the proximal or distal site as indicated in movie. Note the mimic of FUS but only if Etoposide or Arsenite was added to the proximal site whereas distal addition had no effect, thereby strongly suggesting a remote nuclear-axonal crosstalk.

File Name: Supplementary Movie 5
Description: Sames as Supplementary Movie 4 but for lysosomes.

File Name: Supplementary Movie 6
Description: Refers to Fig. 6A & C (showing movie frame at 60 sec of FUS-GFP recruited to Laser cut in MN treated with mimic/rescue compounds). Note the lack of recruitment in the FUS-GFP P525L mutant as opposed to clearly visible fast recruitment followed by slower withdrawal in the FUS-GFP wild type (untreated & Mock). Moreover, note the inhibition of recruitment to the cut (boxed areas) in wild type cells through addition of either PARP1 inhibitor, Etoposide or Arsenite (top gallery). Conversely, PARG, DNA-PK and AdOx inhibitor were each restoring the recruitment-withdrawal cycle in the P525L mutant (bottom gallery).

File Name: Supplementary Movie 7
Description: Same as Supplementary Movie 6 untreated except that NPCs before maturation to MN were used (WT versus mutant P525L FUS-GFP, Table 1 and Supplementary Fig. 1). Note the lack of recruitment to the cut (boxed areas) in Mut cells as opposed to clearly visible fast recruitment followed by slower withdrawal in the FUS-GFP wild type, consistent with matured MN (Fig. 6A, Supplementary Movie 6).

File Name: Supplementary Movie 8
Description: Refers to Fig. 6G (showing maximum intensity projections of Mitotracker JC-1, left):

1. PARP1 inhibitor addition to Ctrl either exclusively to the proximal or distal site as indicated in movie.
Note the mimic of FUS Mock but only if PARP1 inhibitor was added to the proximal site whereas distal addition had no effect, thereby strongly suggesting a remote nuclear-axonal crosstalk.

2. PARG inhibitor addition to FUS either exclusively to the proximal or distal site as indicated in movie.
Note the rescue of FUS as compared to Ctrl Mock but only if PARG inhibitor was added to the proximal site whereas distal addition had no effect, again suggesting a remote nuclear-axonal crosstalk.

3. DNA-PK or AdOx inhibitor addition to FUS exclusively to the proximal site. Note the rescue of FUS as compared to Ctrl Mock, again suggesting a remote nuclear-axonal crosstalk.

File Name: Supplementary Movie 9
Description: Refers to Fig. 6G (showing maximum intensity projections of Lysotracker DND-99, right). Same as Supplementary Movie 8 but for lysosomes.

File Name: Supplementary Movie 10
Description: Refers to Fig. 6M, left column (showing maximum intensity projections of Mitotracker Deep Red FM, LUT Red Hot, and FUS-GFP at Laser cut in green). Shown is the impact of microtubule disruption (24 hrs Nocodazole, 5µM, mid) or respiratory inhibition (24 hrs Oligomycin A, 10µM, bottom) on the recruitment of WT FUS-GFP to the Laser cut in nuclei (boxed areas) in uncompartmentalized MN. Note the unaltered FUS recruitment despite the severe disruption of the mitochondria network along with loss of processive motility in the treated cells.

File Name: Supplementary Movie 11
Description: Refers to Fig. 6M, mid and right column (showing maximum intensity projections of Mitotracker Deep Red FM, LUT Red Hot, and FUS-GFP at Laser cut in green). Shown is the impact of microtubule disruption (24 hrs Nocodazole, 5µM, mid) or respiratory inhibition (24 hrs Oligomycin A, 10µM, bottom) exclusively at the distal site on the recruitment of WT FUS-GFP to the Laser cut in nuclei (boxed areas) at the proximal seeding site in MFCs. Note the unaltered FUS recruitment (proximal) despite the severe distal disruption of the mitochondria network along with loss of processive motility in the distally treated cells.

File Name: Supplementary Movie 12
Description: Refers to Fig. 7B (showing movie frame at 60 sec of FUS-GFP wild type recruited to Laser cut in MNs treated with mimic compounds). Note the inhibition of recruitment already 2 hrs

after addition of PARP1 inhibitor (i.e. long before loss of nuclear FUS as shown in Fig.6A) whereas Etoposide required a longer incubation time beyond 6 hrs before efficient inhibition of FUS-GFP recruitment to the Laser cut occurred (i.e. after loss of nuclear FUS as shown in Fig. 7A).

File Name: Supplementary Movie 13

Description: Refers to Fig. 7D (showing movie frame at 60 sec of FUS-GFP recruited to Laser cut in MNs double-treated 24 hrs with compounds). Note the inhibition of recruitment to the cut (boxed areas) in mutant FUS cells (far left) through double treatment with DNA-PKi and PARP1i whereas DNA-PKi alone leads to a rescue (Supplementary Movie 6), suggesting that PARP1 functions upstream of DNA-PK in DNA damage response. Furthermore, note that inhibition of recruitment through Etoposide or Arsenite in WT control cells (Supplementary Movie 6) was reverted through double treatment with either DNA-PKi or AdOx, suggesting a potent counterbalancing of Etoposide/Arsenite-driven displacement of FUS from the nucleus, thereby rescuing FUS recruitment to the cut (boxed areas).
